# Incidence of Atypical Femoral Fractures in Patients on Osteoporosis Therapy—A Registry‐Based Cohort Study

**DOI:** 10.1002/jbm4.10681

**Published:** 2022-09-22

**Authors:** Judith Everts‐Graber, Harald Bonel, Daniel Lehmann, Brigitta Gahl, HansJörg Häuselmann, Ueli Studer, Hans‐Rudolf Ziswiler, Stephan Reichenbach, Thomas Lehmann

**Affiliations:** ^1^ OsteoRheuma Bern Bern Switzerland; ^2^ Department of Rheumatology and Immunology, Inselspital Bern University Hospital, University of Bern Bern Switzerland; ^3^ Campus Stiftung Lindenhof Bern Swiss Institute for Translational and Entrepreneurial Medicine Bern Switzerland; ^4^ Department of Radiology Lindenhof Hospital Bern Switzerland; ^5^ Department of Radiology Inselspital, University of Bern Bern Switzerland; ^6^ University of Bern, Faculty of Medicine Bern Switzerland; ^7^ Clinical Trial Unit University of Bern Bern Switzerland; ^8^ Zentrum für Rheuma‐ und Knochenerkrankungen Hirslanden Zürich Switzerland; ^9^ Institute for Social and Preventive Medicine University of Bern Bern Switzerland

**Keywords:** AFF, ATYPICAL FEMORAL FRACTURES, BISPHOSPHONATES, DENOSUMAB, OSTEOPOROSIS

## Abstract

Atypical femoral fractures (AFFs) have been reported in patients taking bisphosphonates (BPs) for osteoporosis therapy but also in patients with no exposure to these drugs. In contrast, less is known about the incidence of AFFs in patients taking denosumab. This registry‐based cohort study analyzed the incidence of AFFs in patients with suspected or confirmed osteoporosis who were included in the osteoporosis register of the Swiss Society of Rheumatology between January 2015 and September 2019. Statistical analyses included incidence rates, rate ratios, and hazard ratios for AFFs, and considered sequential therapies and drug holidays as time‐dependent covariates. Among the 9956 subjects in the cohort, 53 had subtrochanteric or femoral shaft fractures. Ten fractures occurred under BP or denosumab treatment and two under teriparatide therapy. Five fractures were classified as AFFs based on the revised American Society of Bone and Mineral Research case definition of AFFs from 2014. Three AFFs occurred in women being treated with denosumab at the time of diagnosis, all with prior BP use (10, 7, and 1 years, respectively). One AFF developed in a woman receiving ibandronate and one arose in a woman receiving glucocorticoids rather than antiresorptive therapy. The incidence of AFFs per 10,000 observed patient‐years was 7.1 in patients receiving denosumab and 0.9 in patients with BP‐associated AFFs, yielding a rate ratio of 7.9 (95% confidence interval [CI] 0.63–413), *p* = 0.073. The risk of AFFs was not significantly higher in patients receiving denosumab therapy compared with BP therapy (hazard ratio = 7.07, 95% CI 0.74–68.01, *p* = 0.090). We conclude that the risk of AFFs is low in patients taking BPs, denosumab, or both sequentially. All three patients with AFFs under denosumab therapy had undergone prior BP therapy. © 2022 The Authors. *JBMR Plus* published by Wiley Periodicals LLC on behalf of American Society for Bone and Mineral Research.

## Introduction

Antiresorptive therapies such as bisphosphonates (BPs) and denosumab reduce the risk of fragility fractures, which are major causes of disability and high healthcare costs. However, concerns have been raised about the potential for adverse effects such as atypical femoral fractures (AFFs). Unusual fragility fractures of the femur in patients receiving oral BPs were first described in 2005, followed by case series reporting a strong association with BP therapy. Since then, AFFs have caused uncertainty among patients and their physicians and led to a decline in oral BP use in the United States between 2008 and 2012.^(^ ^1^ ^)^ The American Society for Bone and Mineral Research (ASBMR) convened an international, multidisciplinary task force in 2009 to develop a case definition and identify future areas of research.^(^ ^2^ ^)^ The ASBMR criteria for AFF diagnosis were redefined in 2014 to emphasize the specific radiologic features of AFFs to distinguish them from ordinary fragility fractures of the femoral shaft.^(^ ^3^ ^)^

The pathogenesis of AFFs remains poorly understood, but the long‐term suppression of bone resorption with prolonged use of BPs, leading to micro‐crack propagation and a stress reaction of the lateral femur cortex, has been well explored.^(^ ^4^ ^)^ Other risk factors include lateral femoral bowing and varus hip geometry,^(^ ^5^, ^6^ ^)^ as well as obesity and glucocorticoid use.^(^ ^7^ ^)^ Asians are more susceptible to AFFs than other ethnicities.^(^ ^7^, ^8^ ^)^ Notably, AFFs have also been described in patients who have not been treated with bone‐modifying agents, and the strength of the association between these drugs and AFFs remains controversial.^(^ ^9^ ^)^

The incidence of AFFs in patients who have received BPs for <3 years is estimated to be 0.56 per 10,000 patient‐years compared with 13.1 per 10,000 patient‐years in those treated for >8 years, with a direct relationship between duration of BP exposure and risk of AFF.^(^ ^7^ ^)^ Less is known about AFFs in patients receiving denosumab or sequential treatment with BPs and denosumab (in either order). Although several case series^(^ ^10^, ^11^, ^12^, ^13^, ^14^, ^15^, ^16^, ^17^, ^18^ ^)^ and three randomized controlled trials^(^ ^19^, ^20^, ^21^ ^)^ reported that patients exposed to denosumab developed AFFs, no clear relationship has been established in case–control or cohort studies. The aim of this registry‐based cohort study was to analyze the incidence of AFFs in patients treated with BP therapy, denosumab, or both sequentially in a real‐world setting. We recently analyzed the risk of osteonecrosis of the jaw (ONJ) in patients who received BPs, denosumab, or both sequentially.^(^ ^22^ ^)^ The incidence and risk of ONJ was higher in patients under denosumab compared with BPs (hazard ratio = 3.5, 95% confidence interval (CI) 1.2–10.5, *p* = 0.026). Of note, 9 of the 12 patients who developed ONJ under denosumab treatment received prior BP therapy. In the present study, we analyzed the incidence of AFF in the same study population.

## Materials and Methods

### Study design

This study was conducted at a single non‐academic outpatient center in Switzerland, named OsteoRheuma Bern. Patients reviewed in this cohort study were included in a national register for osteoporosis maintained by the Swiss Society of Rheumatology (https://osteorheuma.ch/top), which has been described before.^(^ ^22^ ^)^ Eligible cohort members were subjects followed from January 1, 2015, to September 30, 2019, who were included in the registry because of suspected osteoporosis because of fragility fractures and/or because they had risk factors for osteoporosis. Retrospective data about past fractures and anti‐osteoporotic therapies were collected in detailed and structured interviews with the patients and verified by consulting the referral information from each patient's corresponding general practitioners, as were prospective data after cohort entry. All subjects underwent at least one dual‐energy X‐ray absorptiometry (DXA) scan and were usually followed up every 2 to 3 years depending on their individual fracture risk and therapeutic strategy. Anti‐osteoporotic drug therapy was initiated in cases of fragility fracture or high fracture risk. The choice of medication was at the discretion of the treating physician, with certain constraints stipulated by the health authorities.

The study was approved by the Ethics Committee of the Canton of Bern, Switzerland (KEKBE 2019–01037), and all subjects provided written informed consent.

### Outcomes

The primary outcome was the incidence of AFFs. We analyzed recorded femoral fractures in all subjects regardless of anti‐osteoporotic therapy. Radiographs and data on clinical circumstances were collected for all subtrochanteric and femoral shaft fractures. Finally, radiographs of these fractures were independently adjudicated by two radiologists who were unaware of the type of antiresorptive treatment, using the revised ASBMR criteria for AFFs from 2014.^(^ ^3^ ^)^ Secondary outcomes were additional risk factors for AFFs and the clinical characteristics and long‐term outcomes of subjects with AFFs.

### Statistical analyses

We analyzed the association of BP and denosumab with the risk of AFFs in a time‐to‐event manner, including both treatments as time‐varying covariates in a Cox regression model. Because of the small number of events, no further covariates could be included in the model.^(^ ^23^ ^)^ We also calculated event rates and standard errors per 10,000 patient years on a log scale, which we back‐transformed into rates with 95% CIs, together with rate ratios. Continuous variables are presented as mean ± standard deviation or as median and interquartile range [IQR]. Categories are presented as number and percentage. Statistical analyses were carried out using Stata 16 (StataCorp, College Station, TX, USA) and *R* was used for the line plot (ggplot2 3.3.5).

## Results

### Study cohort

The study cohort included 9956 subjects enrolled between January 1, 2015 (the implementation date of the osteoporosis register of the Swiss Society of Rheumatology), and September 30, 2019. A total of 6821 subjects received no specific anti‐osteoporotic drug apart from hormone replacement therapy and/or calcium and vitamin D supplements, while 3135 patients received BPs, denosumab, selective estrogen receptor modulators (SERMs), and/or teriparatide. Of these treated patients, 3068 received BPs, denosumab, or both sequentially, and their characteristics are shown in Table 1. This table is similar to Table 1 in Everts‐Graber and colleagues,^(^ ^22^ ^)^ as it refers to the same study population.

**Table 1:** Patient Characteristics by Treatment

	BP only (*n* = 1802)	Dmab only (*n* = 422)	Both (*n* = 844)	*p* Value
Male	271 (15%)	24 (5.7%)	34 (4.0%)	**<0.001**
Age (years)	69 ± 10	69 ± 10	70 ± 8.9	0.18
BMI (kg/m^2^)	25 ± 4.8	24 ± 4.8	24 ± 4.1	**<0.001**
Premenopausal	59 (3.3%)	16 (3.8%)	25 (3.0%)	0.65
Family history of osteoporosis	206 (11%)	40 (9.5%)	81 (10%)	0.26
Use of glucocorticoids (≥5 mg/d for ≥3 mo)	239 (13%)	21 (5.0%)	47 (5.6%)	**<0.001**
Prostate cancer with hormone ablative therapy	4 (0.22%)	2 (0.47%)	2 (0.24%)	0.53
Use of aromatase inhibitors	32 (1.8%)	57 (14%)	46 (5.5%)	**<0.001**
Use of antiepileptic medication	10 (0.55%)	1 (0.24%)	4 (0.47%)	0.87
Rheumatoid arthritis	87 (4.8%)	8 (1.9%)	27 (3.2%)	**0.007**
Axial spondylarthritis	10 (0.55%)	1 (0.24%)	1 (0.12%)	0.26
Immobility/need for a walking aid	95 (5.3%)	28 (6.6%)	33 (3.9%)	0.10
Type 1 diabetes	20 (1.1%)	6 (1.4%)	4 (0.47%)	0.15
Chronic obstructive pulmonary disease	66 (3.7%)	10 (2.4%)	17 (2.0%)	0.050
Hypogonadism in males	11 (0.61%)	1 (0.24%)	1 (0.12%)	0.17
Early menopause in females (<45 years)	109 (6.0%)	29 (6.9%)	51 (6.0%)	0.79
Primary hyperparathyroidism	16 (0.89%)	4 (0.95%)	4 (0.47%)	0.45
Current smoking	184 (10%)	45 (11%)	52 (6.2%)	**0.001**
Alcohol intake >30 g/d	30 (1.7%)	2 (0.47%)	2 (0.24%)	**0.001**
*T*‐score lumbar spine	−1.8 ± 1.4	−2.3 ± 1.5	−2.4 ± 1.3	**<0.001**
*T*‐score femoral neck	−2.1 ± 0.73	−2.2 ± 0.79	−2.2 ± 0.73	**<0.001**
*T*‐score total hip	−1.8 ± 1.2	−1.9 ± 0.92	−1.9 ± 0.84	**0.009**
*T*‐score 1/3 radius	−2.2 ± 1.4	−2.7 ± 1.4	−2.2 ± 1.6	0.18
*T*‐score minimum	−2.5 ± 1.2	−2.8 ± 0.98	−2.8 ± 0.84	**<0.001**
Trabecular bone score	1.2 ± 0.16	1.2 ± 0.15	1.2 ± 0.17	**0.023**
Vertebral fracture (s)	534 (30%)	126 (30%)	264 (31%)	0.68
Hip fracture (s)	93 (5.2%)	17 (4.0%)	36 (4.3%)	0.49
Nonvertebral fracture (s)	494 (27%)	100 (24%)	244 (29%)	0.14
Duration of BP treatment (months)	40 [29 to 64]	0.0 [0.0 to 0.0]	44 [12 to 75]	**<0.001**
Duration of Dmab treatment (months)	0.0 [0.0 to 0.0]	37 [26 to 58]	32 [27 to 56]	**<0.001**

BMI = body mass index; BP = bisphosphonate; Dmab = denosumab.

Continuous variables: median with interquartile range [IQR]. Categorical variables: percentages of total of each subgroup.

### Description of AFF cases

**Fig. 1:**
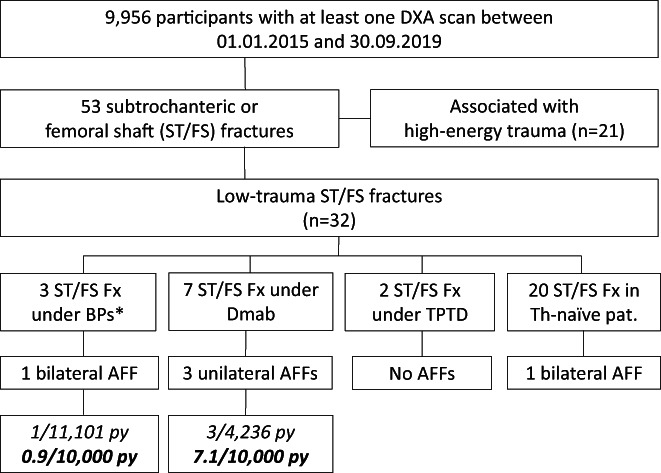
Overview of all subtrochanteric or femoral shaft fractures in the study cohort. AFF = atypical femoral fracture; BPs = bisphosphonates; Dmab = denosumab; py = patient‐years; ST/FS Fx = subtrochanteric or femoral shaft fractures; TPTD = teriparatide; Th = therapy; *One ST/FN fracture occurred after BP discontinuation.

A total of 53 subtrochanteric or femoral shaft fractures were recorded; of these, 21 and 32 were associated with high‐ and low‐energy trauma, respectively (Fig. 1). Ten of the low‐trauma subtrochanteric or femoral shaft fractures occurred under BP or denosumab treatment and two under teriparatide therapy. Eight did not meet the criteria for AFF (ie, they were periprosthetic or comminuted fractures or had no substantially transverse configuration). Four were adjudicated as AFFs: Three unilateral AFFs occurred in women being treated with denosumab, all with prior BP use (10, 7, and 1 years, respectively), and one bilateral AFF developed in a woman receiving ibandronate. In addition, one bilateral AFF arose in a woman receiving glucocorticoids rather than antiresorptive therapy. There was 100% agreement between the two radiologists. Of note, none of the patients had both an AFF and ONJ.

**Fig. 2:**
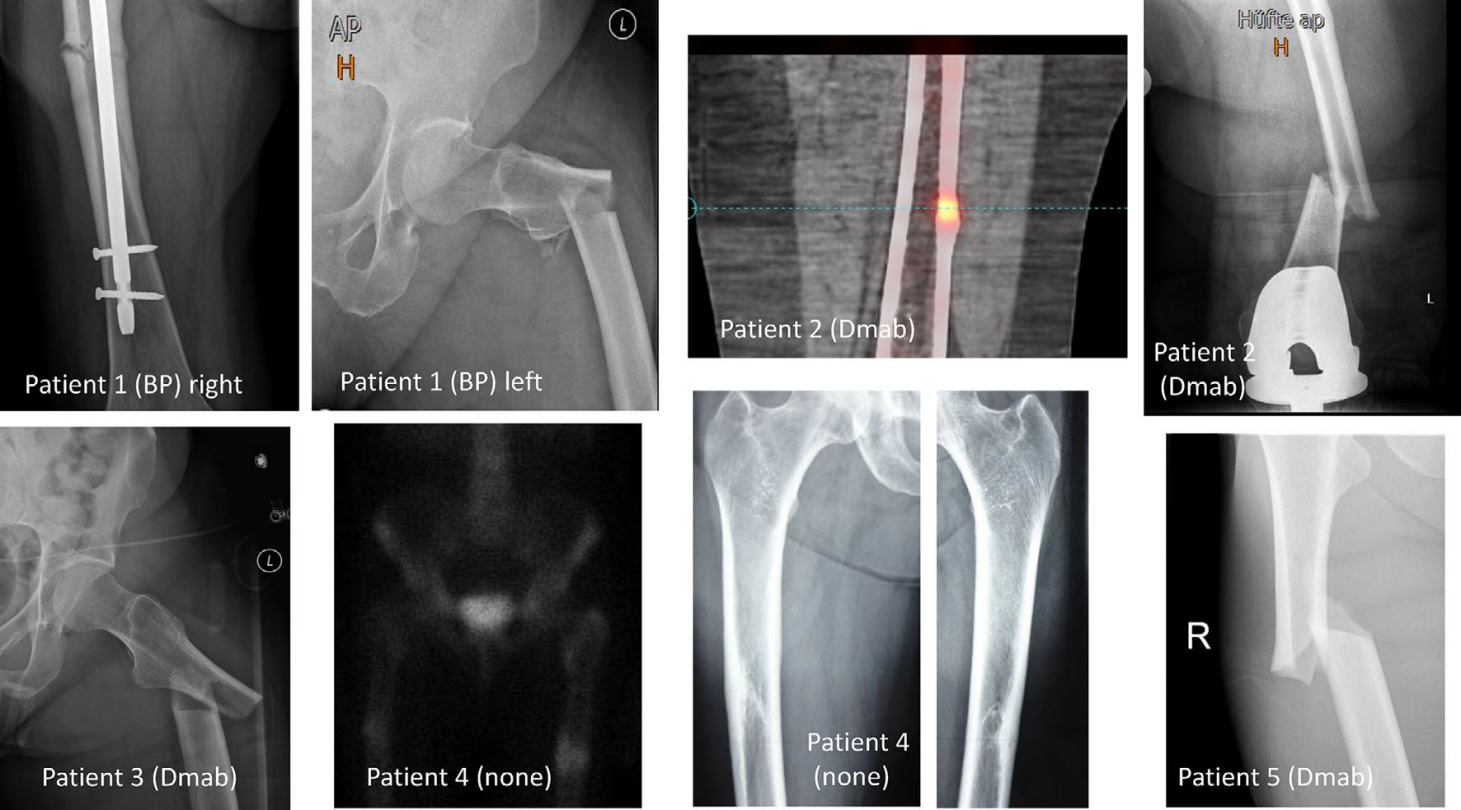
Images of the five patients with atypical femoral fracture. BP = bisphosphonate; Dmab = denosumab.

The clinical characteristics of the 5 patients with AFFs are described in Table 2, and the images of their femora are depicted in Fig. 2. The specific circumstances and the long‐term outcomes of these patients are as follows:

**Table 2:** Clinical Characteristics of Patients with AFFs

	Age^a^ (years), sex	*T*‐scores^b^ (LS/FN)	Prior Fx	Prior Th	Additional risk factors	AFF	Ongoing course
1	77, f	−1.1/na	Radius (72 yr)	ALN/IBN 5 yr	Severe osteoarthritis	Complete bilateral (within 3 months)	VFx (Th12) 5 months after AFF, Th with TPTD. Periprosthetic femoral Fx 14 months later, Dmab was added to TPTD.
2	66, f	−1.3/−2.7	Metatarsale (6x)	ZOL 1 yr, Dmab 2.5 yr	Polyarthritis, repetitive low‐dose GC	Complete unilateral	ZOL after Dmab, pubic and sacral fractures after 2.5 years, later TPTD and ZOL
3	82, f	−2.3/−2.0	Humerus (79 yr)	ALN 10 yr, 2 yr pause, Dmab 3 yr	None	Complete unilateral	Dmab stopped, Th12 fracture 2 yr later, ZOL
4	48, f	−2.8/−2.5	None	None	Sarcoidosis, low‐dose GC	Bilateral (complete and incomplete)	Repeated vertebral fractures, Th with IBN; later Dmab and Dmab/TPTD
5	66, f	−2.5/−2.7	None	ALN 7 yr, Dmab 6 yr	None	Complete unilateral	ZOL after Dmab, no further Fx

ALN = alendronate; Dmab = denosumab; f = female; Fx = fracture; GC = glucocorticoids; IBN = ibandronate; na = not available; Th = therapy; TPTD = teriparatide; ZOL = zoledronate.

^a^ Age at AFF.

^b^ *T*‐scores before AFF.

Patient 1: A 77‐year‐old woman with severe osteoarthritis experienced a complete AFF on the left side and 3 months later developed another on the right side. Her prior therapy for postmenopausal osteoporosis with a history of a distal radius fracture was oral alendronate and oral ibandronate for 4 years. Five months after the second AFF and after discontinuation of ibandronate therapy, she sustained a Th12 fracture. Teriparatide was started, and 14 months later, a periprosthetic femoral shaft fracture right was diagnosed. This fracture demonstrated several similarities to an AFF (prior groin pain, thickening of the lateral cortex, and a transverse fracture line). Denosumab was added to the teriparatide treatment, and no further fractures occurred.

Patient 2: A 66‐year‐old woman with undifferentiated polyarthritis was treated repetitively with low‐dose glucocorticoids and with zoledronate due to osteoporosis. She suffered multiple metatarsal fractures and her serum alkaline phosphatase levels were repeatedly low. Genetic testing for hypophosphatasia was negative. Zoledronate was switched to denosumab after 1 year, and she sustained an AFF 2.5 years later. Although the AFF was first diagnosed by single‐photon emission computed tomography (SPECT) as metabolically active thickening of the lateral cortex, the fracture became complete after 5 weeks. Denosumab was switched to zoledronate and later to teriparatide after pubic and sacral fractures occurred.

Patient 3: An 82‐year‐old woman was treated with alendronate for 10 years and, after a 2‐year drug holiday, with denosumab for 3 years due to postmenopausal osteoporosis and a prior humeral fracture. She suffered a complete AFF while falling but reported a prior leg pain. Denosumab was stopped, and zoledronate was started 2 years later after diagnosis of a morphometric Th12 fracture. No further fractures occurred within the next 5 years.

Patient 4: This postmenopausal, 48‐year‐old woman was diagnosed with sarcoidosis with bilateral hilar lymphadenopathy and a history of glucocorticoid therapy. In addition, she experienced malabsorption after gastrectomy for gastric adenoma. Her serum vitamin D and calcium levels were in the normal range under supplemental therapy. Her serum tryptase levels were consistently slightly elevated, but bone biopsy was normal without signs of systemic mastocytosis. Because of increasing groin pain, radiographs of the pelvis and femora were performed. These showed thickening of the cortical bone bilaterally, and a diagnosis of AFF was confirmed by bone scintigram. One month later, the patient sustained a complete femoral fracture on the right side, which was treated with intramedullary nailing. Antiresorptive therapy with intravenous ibandronate was started 3 months later but was stopped after 16 months because of incomplete healing of the AFF. Six months after ibandronate discontinuation, the patient sustained two vertebral fractures (L_1_ and L_2_) and teriparatide was initiated. One year later, a Th11 fracture occurred and denosumab was added to the teriparatide regimen.

Patient 5: A 66‐year‐old woman was diagnosed with postmenopausal osteoporosis (initial *T*‐scores unknown) and was treated with alendronate for 7 years and then with denosumab for another 6 years. She reported femoral pain when walking, and a few weeks later a complete AFF occurred and was treated surgically with intramedullary nailing. Denosumab was switched to zoledronate, and no further fractures occurred within the next 2 years.

### Description of sequential therapies and drug holidays

**Fig. 3:**
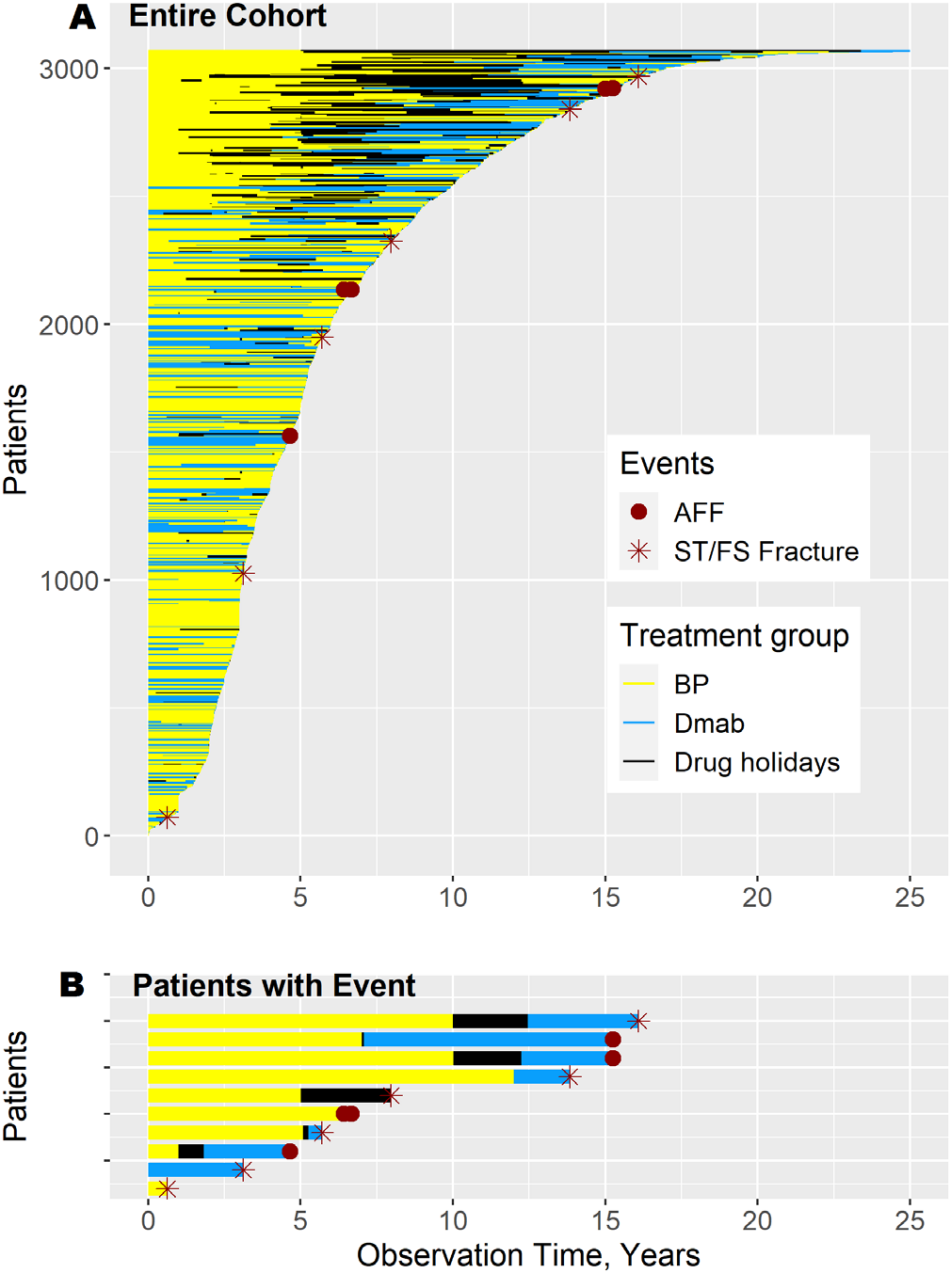
Line plot of the study cohort. BP = bisphosphonate; Dmab = denosumab; AFF = atypical femoral fracture; ST/FS = subtrochanteric or femoral shaft. (*A*) This line plot represents the drug exposures of all patients who received BPs and/or denosumab (*n* = 3068). Each line represents the treatment and drug holiday sequences of one patient (BPs: yellow; denosumab: light blue; drug holiday: black), and the patients were sorted by total observation time. The stars indicate typical subtrochanteric or femoral shaft fractures (ST/FS Fx) and the dots represent AFFs. In each case, the end of observation was the patient's last visit before the end of the study (September 30, 2019). (*B*) Line plot of selected patients who sustained a subtrochanteric or femoral shaft fracture or an AFF.

Of 3068 subjects who received BP and/or denosumab therapy, 2646 were treated with BPs and 1266 with denosumab. We analyzed 11,101 observed patient‐years for BP therapy (48% oral BPs and 52% intravenous BPs) and 4236 patient‐years for denosumab therapy. Oral BPs were alendronate (70 mg/wk) and ibandronate (150 mg/mo), while intravenous BPs were ibandronate (3 mg q3 mo) and zoledronate (5 mg/yr). Denosumab was administered at 60 mg q6 mo. A total of 844 patients (28%) received sequential therapies (first a BP and then denosumab, or vice versa) with or without drug holidays. The therapy sequences and drug holidays of all patients are depicted in a line plot in Fig. 3*A*. This plot shows that many patients who were initially treated with a BP switched to denosumab, and most patients who discontinued denosumab received subsequent BP therapy. Most AFFs occurred after long‐term antiresorptive therapy, which was not the case for regular subtrochanteric or femoral shaft fractures (Fig. 3*B*). Overall, drug holidays comprised only a small proportion of the observation time (2614 patient‐years; 15%). In total, 1048 (34%) patients had a drug holiday, with a median duration of 1.9 years [0.5 to 3.6]. Regarding long‐term therapy, 471 patients had a cumulative antiresorptive treatment duration of ≥8 years, while in 212 patients, this treatment was uninterrupted.

### Incidence rates and risks of AFFs with specific therapies

Among 3068 patients receiving BP and/or denosumab therapy, one experienced an AFF under BP therapy and three under denosumab, yielding incidence rates per 10,000 patient‐years of 0.90 (95% CI 0.13–6.40) for BPs and 7.08 (2.28–21.96) for denosumab. The rate ratio between denosumab and BPs was 7.86 (0.63–413, *p* = 0.073) (Table 3*A*). All three patients who developed AFFs while receiving denosumab had undergone prior therapy with BPs. In an analysis of hazard ratios with treatment types as time‐dependent covariates, the risk of AFFs was not significantly higher under denosumab therapy compared with BP treatment (hazard ratio 7.07, 95% CI 0.74–68.01, *p* = 0.090) (Table 3*B*). Of note, one patient sustained a bilateral AFF in the absence of antiresorptive drugs. This AFF was not considered in the statistical model.

**Table 3:** Incidence Rates of Atypical Femoral Fractures (AFFs)

(*A*) AFF event rates			
Treatment	Patient‐years	No. of events^a^	Rate per 10,000 patient‐years (95% CI)
BP	11,101	1	0.90 (0.13 to 6.40)
Dmab	4236	3	7.08 (2.28 to 21.96)
Drug holidays	2614	0	0.00 (0.00 to 0.001)

(*B*) Risk of AFF by treatment		
Treatment	Hazard ratio (95% CI)	*p* Value
BP	Reference	
Dmab	7.17 (0.75 to 69)	0.088

Rate ratio of Dmab versus BP: 7.86 (0.63 to 413, *p* = 0.073).

Incidence rates (*A*) and hazard ratios (*B*) of AFF. With bisphosphonates (BPs) as the reference, denosumab (Dmab) is not significantly associated with a higher risk of AFF.

^a^ Note that one AFF occurred in a patient without antiresorptive therapy.

## Discussion

### AFFs under sequential antiresorptive treatment

In this cohort study, we analyzed the incidence and risk of AFFs in an osteoporosis registry in Switzerland. A total of 9956 subjects with or without anti‐osteoporotic therapies were included, and AFFs were observed in one woman under ibandronate, in three women under denosumab (all with prior BP use), and in one woman without antiresorptive therapy. We report a numerically (but not significantly) higher incidence of AFFs in patients under denosumab therapy (three cases, incidence of 7.07 per 10,000 patient years) than in those who received only BPs (one case, 0.9/10,000 patient years), but all three patients who developed AFFs during denosumab treatment had been pretreated with BPs, for 10, 7, and 1 years, respectively. One of these women received low‐dose glucocorticoid therapy, which has been identified as a risk factor for AFFs.^(^ ^24^, ^25^, ^26^ ^)^

There are only limited data on AFFs associated with denosumab use or under sequential therapies with BPs and denosumab. Several case reports and series reported AFFs under denosumab treatment, both in patients with the conventional 60‐mg dose for osteoporosis and in those with higher oncological doses. Importantly, the majority of these studies documented prior BP exposure,^(^ ^11^, ^13^, ^14^, ^21^ ^)^ and only four cases of AFFs in BP‐naïve patients with osteoporosis have been reported. Two cases were identified in the FREEDOM extension study (Fracture Reduction Evaluation of Denosumab in Osteoporosis Every 6 Months) (0.8 per 10,000 participant‐years), one occurring in the long‐term denosumab group after 7 years of exposure and one in the crossover group in year 3 of denosumab therapy.^(^ ^27^ ^)^ One of these patients achieved fracture healing within 6 months of stopping denosumab and the other patient continued denosumab, but no information about fracture healing is available. Additionally, one BP‐naïve patient in a randomized controlled trial of denosumab in patients with glucocorticoid‐induced osteoporosis developed an AFF after the second dose of denosumab, but she had been exposed to glucocorticoids for more than 30 years.^(^ ^19^ ^)^ Finally, one patient with bilateral incomplete AFFs after 3 years of denosumab therapy healed with conservative management and denosumab discontinuation (without subsequent therapy).^(^ ^18^ ^)^

These observations raise the question of how treatment sequences involving denosumab and BPs affect the risk of AFFs. AFFs under BP therapy usually occur after treatment durations of ≥8 years.^(^ ^7^ ^)^ Interestingly, one randomized controlled trial and several case reports of AFFs under denosumab demonstrated shorter cumulative antiresorptive treatment durations, and notably, some patients had no extensive prior BP therapy.^(^ ^10^, ^18^, ^21^ ^)^ On long‐term bisphosphonate therapy, AFFs are associated with a duration‐dependent increase in mean degree of mineralization of bone, leading to a significantly higher mean degree of mineralization than the non‐AFFs with similar exposure to bisphosphonates and low remodeling assessed by histomorphometry.^(^ ^28^ ^)^ Because bisphosphonates have high affinity to bone mineral and lining the walls of the osteocyte lacunae, the accumulation of matrix‐bound bisphosphonates could reduce the response of the cytoskeleton to mechanical strains.^(^ ^28^, ^29^ ^)^ Hence, AFFs are presumably not only the consequence of long‐term suppression of bone remodeling, as the rate of AFFs in patients with denosumab treatment for 10 years was very low.^(^ ^27^ ^)^ AFFs were also reported in patients taking romosozumab^(^ ^30^ ^)^ or odanacitinib.^(^ ^31^, ^32^ ^)^ Although all of these drugs act at least in part by inhibiting bone resorption, the sequential use of first bisphosphonates, followed by highly active antiresorptive agents may lead to earlier AFF onset than with long‐term BP monotherapy. In this study, one patient developed an AFF under denosumab after receiving antiresorptive therapy for a total of <5 years. Although prior BP therapy may play an important role in the onset of this AFF, it cannot solely be attributed to the history of BP use. The risk of AFF decreases rapidly within 1 year after BP discontinuation,^(^ ^7^, ^25^ ^)^ suggesting that subsequent denosumab therapy plays a critical role in the development of AFF. Transitioning from BPs to a more potent antiresorptive could prevent the clearance of accumulated microcracks from bone matrix and increase the risk of AFF.^(^ ^33^ ^)^

In the same population as that analyzed in this study, we recently reported a higher risk of ONJ under denosumab compared with BPs.^(^ ^22^ ^)^ In addition, the sequential use of BPs followed by denosumab has been discussed as a possible risk factor for ONJ development in patients with cancer‐related ONJs.^(^ ^34^ ^)^ However, while we identified 17 patients who developed ONJ while receiving BPs or denosumab, only four sustained an AFF. One explanation for this discrepancy may be the real‐world study population in which these rare adverse events were examined. Patient comorbidities might increase the risk of ONJ to a greater extent than AFF. It is also possible that sequential therapy with BPs and denosumab may itself be the cause, perhaps predisposing individuals to ONJ more than to AFF. On the other hand, this discrepancy in the incidence of ONJ and AFF was not observed in four randomized controlled trials in patients who received BPs followed by denosumab treatment.^(^ ^35^ ^)^

### Management of AFFs under denosumab

The long‐term course of our patients who suffered an AFF demonstrates the difficulty of managing these fractures. The discontinuation of antiresorptive therapy after an AFF is strongly recommended, in particular to prevent a contralateral AFF and to facilitate adequate fracture healing.^(^ ^3^ ^)^ On the other hand, prevention of subsequent fractures is necessary, as these patients are usually at high risk of fragility fractures.^(^ ^36^ ^)^ If an AFF occurs under BP therapy, the medication can be paused and then reinitiated after complete fracture healing if the patient has a high fracture risk. In contrast to BPs, discontinuation of denosumab without subsequent antiresorptive therapy is strongly disadvantageous, as this can lead to a rebound effect with an increased risk of multiple vertebral fractures.^(^ ^37^ ^)^ Some observational studies demonstrated that surgically treated AFFs healed faster if BP therapy was switched to teriparatide.^(^ ^38^ ^)^ However, there is insufficient evidence as to whether this is also the case in AFFs experienced under denosumab, and subsequent teriparatide after denosumab can result in progressive or transient bone loss.^(^ ^39^ ^)^ In a systematic review by the European Calcified Tissue Society, two patients developed a second complete AFF on continued denosumab and a third patient had bilateral, recurrent, incomplete AFFs despite the use of teriparatide.^(^ ^38^ ^)^ Van de Laarschot and colleagues therefore proposed a short course of BP or SERM therapy after denosumab discontinuation in patients with bilateral surgically treated AFFs and in those with unilateral surgically treated AFF and no radiological signs of incomplete AFF involving the contralateral femur.^(^ ^38^ ^)^ Indeed, in two patients with AFFs under denosumab therapy in this study, switching from denosumab to a BP was not associated with the development of an AFF in the contralateral femur.

### Limitations

Our observations have a few limitations. First, the study population was small for analysis of a rare adverse event, and the confidence intervals of incidence rates and the risk of AFFs were therefore extended. However, this study cohort was large enough to demonstrate significant differences between the risk of ONJ under denosumab compared with BPs.^(^ ^22^ ^)^ Another limitation is the fact that nearly half of the antiresorptive therapies were recorded retrospectively. Missing or false information cannot formally be excluded. However, these retrospective data were obtained directly from patients and from data provided by the patients' general practitioners. Further, 10% of our patients with BP or denosumab therapy received glucocorticoids at some point. This combination of medications has been identified as a risk factor for AFFs in several studies.^(^ ^7^, ^24^, ^40^ ^)^ The most important strength of this cohort study was the assessment of both radiographs and clinical settings of all patients with subtrochanteric or diaphyseal femoral fractures. Most previous cohort studies relied on non‐specific ICD coding instead of radiograph evaluation.^(^ ^41^ ^)^ In this context, it is noteworthy that two of the five AFFs in our cohort were initially diagnosed as common subtrochanteric fractures, and one incomplete AFF was misclassified as a suspected benign bone tumor on SPECT images.

In our cohort of patients with osteoporosis, the risk of AFFs was low in those taking BPs, denosumab, or both sequentially. All three patients who developed AFFs while under denosumab had received prior BP therapy. In two of these patients, switching to a BP after denosumab discontinuation was not associated with the development of a contralateral femoral fracture. However, a larger cohort with longer observational periods is required to examine the impact of sequential antiresorptive therapies on the risk of AFF. In addition, the optimal therapy after an AFF that occurs under denosumab should be further investigated.

## Disclosures

JE‐G, DL, BG, SR, and TL have nothing to declare and have no conflicts of interest. HB received consultancy fees from Novartis. HJH received occasional speaker's fees from Amgen, Sandoz, Eli Lilly, and Labatec. H‐RZ received consultancy fees from Abbvie, Celgene, Amgen, and Mylan/Viatris. US received congress and travel expenses from Sandoz, Pfizer, and Janssen Pharmaceutica, and consultancy fees from Novartis and Amgen.

## Author Contributions

**Judith Everts:** Conceptualization; data curation; formal analysis; funding acquisition; investigation; methodology; project administration; supervision; writing – original draft; writing – review and editing. **Harald Bonel:** Conceptualization; formal analysis; funding acquisition; investigation; supervision; validation; writing – review and editing. **Daniel Lehmann:** Data curation; formal analysis; investigation; writing – review and editing. **Brigitta Gahl:** Conceptualization; formal analysis; investigation; methodology; software; validation; writing – original draft; writing – review and editing. **Hans‐Joerg Häuselmann:** Data curation; funding acquisition; investigation; project administration; supervision; writing – original draft; writing – review and editing. **Ueli Studer:** Data curation; funding acquisition; investigation; writing – review and editing. **Hans Rudolf Ziswiler:** Data curation; funding acquisition; investigation; writing – review and editing. **Stephan Reichenbach:** Conceptualization; formal analysis; funding acquisition; methodology; project administration; supervision; writing – original draft; writing – review and editing. **Thomas Peter Lehmann:** Conceptualization; data curation; formal analysis; funding acquisition; investigation; project administration; supervision; writing – original draft; writing – review and editing.

### Peer Review

The peer review history for this article is available at https://publons.com/publon/10.1002/jbm4.10681.

## Data Availability

The data that support the findings of this study are available from the corresponding author upon reasonable request.
